# The effect of surface treatments and graphene-based modifications on mechanical properties of natural jute fiber composites: A review

**DOI:** 10.1016/j.isci.2021.103597

**Published:** 2021-12-10

**Authors:** Mohammad Hamidul Islam, Md Rashedul Islam, Marzia Dulal, Shaila Afroj, Nazmul Karim

**Affiliations:** 1 Centre for Print Research (CFPR), The University of West of England, Frenchay, Bristol BS16 1QY, UK

**Keywords:** Materials science, Mechanical processing, Nanotechnology fabrication

## Abstract

Natural fiber reinforced composites (FRC) are of great interests, because of their biodegradability, recyclability, and environmental benefits over synthetic FRC. Natural jute FRC could provide an environmentally sustainable, light weight, and cost-effective alternative to synthetic FRC. However, the application of natural jute FRC is limited because of their poor mechanical and interfacial properties. Graphene and its derivatives could potentially be applied to modify jute fiber surface for manufacturing natural FRC with excellent mechanical properties, and lower environmental impacts. Here, we review the physical and chemical treatments, and graphene-based modifications of jute fibers, and their effect on mechanical properties of jute FRC. We introduce jute fiber structure, chemical compositions, and their potential applications first. We then provide an overview of various surface treatments used to improve mechanical properties of jute FRC. We discuss and compare various graphene derivative-based surface modifications of jute fibers, and their impact on the performance of FRC. Finally, we provide our future perspective on graphene-based jute fibers research to enable next generation strong and sustainable FRC for high performance engineering applications without conferring environmental problems.

## Graphical abstract

**Figure:**
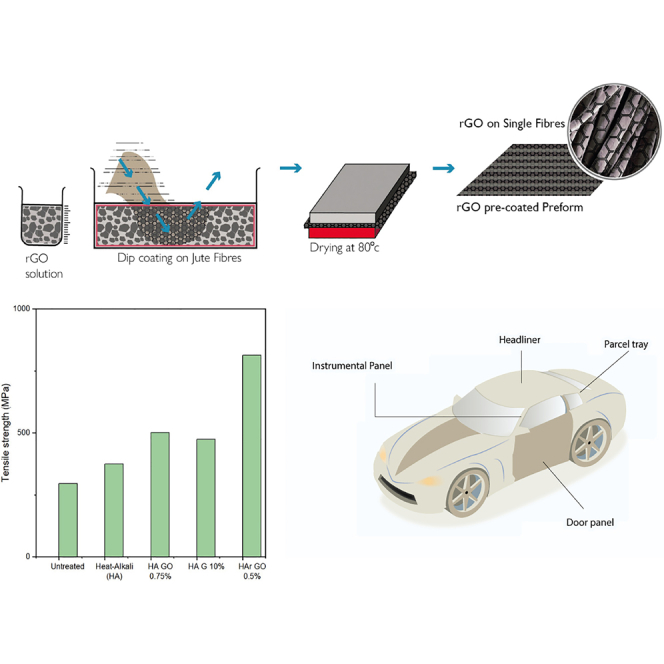

## Introduction

Sustainable and biodegradable natural fiber reinforced composites (FRC) have received significant interests in recent years, because of their lower environmental impacts (Mohanty et al., 2018; Zheng and Suh, 2019; Karim et al., 2015), including less carbon emission and fossil fuel consumption, lower cost and density, and ease of fabrication. The use of such fibers could offer an unprecedented combination of stiffness, strength, and toughness at low density (Wegst et al., 2015; Karim et al., 2021b). Therefore, natural FRC could potentially be used as lightweight and environmentally sustainable composites to replace glass, carbon, or other synthetic FRCs in numerous applications such as automotive, construction, and household. The commonly used natural fibers to manufacture the composite are flax, jute, hemp, banana, ramie, and kenaf. Among them, the jute fibers have attracted significant interest, as jute is the second most produced (mainly in Bangladesh, India, and China) natural fiber after cotton, and is at least 50% cheaper than flax and other natural fibers (Koronis et al., 2013). However, jute fibers suffer from lower mechanical properties and poor adhesion when reinforced with a matrix, because of the presence of large amounts (20 wt.%–50 wt.%) of noncellulosic materials such as hemicellulose and lignin (Sarker et al., 2018). The presence of noncellulosic materials provides lower crystallinity and hydrophilicity of fibers (Gassan and Bledzki, 1999b), and is responsible for poor mechanical properties of composites. Nevertheless, the strong interfacial bond between fiber and matrix could potentially provide higher mechanical and interfacial properties of the composites. The surface modification of the jute fiber is considered to be essential to improve their adhesion with a polymer matrix.

There are various physical and chemical treatments that have been carried out to remove the noncellulosic materials and improve the mechanical properties of the jute fiber and their composites. Among them, the alkali treatment is the most popular surface treatment which removes noncellulosic materials and impurities from the interfibrillar region of jute fiber. Thus such treatment makes the fibrils more capable of rearranging themselves along the direction of tensile deformation and provides a better load sharing capability between themselves to contribute to higher stress development during the tensile test (Bledzki and Gassan, 1999). The surface treatment of jute fiber at lower alkali concentration for a prolonged period of time can enhance the mechanical properties of jute fiber (Roy et al., 2012; Sarker et al., 2018). In addition, various other combined surface treatments including alkali-silane (Dilfi et al., 2018), alkali-plasma (Gibeop et al., 2013), alkali-beaching (Rajesh and Prasad, 2014), and alkali-acetylation (Mwaikambo and Ansell, 1999) have been investigated. However, the improvement of mechanical properties with such treatments is limited and some of the treatments are expensive (such as plasma treatment).

Recently, graphene and its derivatives including graphene flakes (G), graphene oxide (GO), and reduced graphene oxide (rGO) have attracted tremendous attention for high-performance composite applications because of their incredible mechanical properties. Graphene derivatives (GO and rGO) could be produced in a huge quantity in their stable dispersions. In addition, such materials provide good chemical reactivity and handling characteristics because of their intrinsic functional groups (Afroj et al., 2019). Furthermore, graphene-based and electrically conductive flakes can be produced in a scalable quantity *via* microfluidization technique, and used for smart composites applications (Karim et al., 2018; Afroj et al., 2020). Previous studies (Da Luz et al., 2020, Karim et al., 2021b; Sarker et al., 2018; Sarker et al., 2019) demonstrate significant improvement in mechanical properties and performances of graphene-modified jute fibers and their composites *via* forming either bonding (GO) or mechanical interlocking (G) between fibers and graphene-based flakes. In addition, graphene-based jute FRC have been developed for high-performance composites multifunctional smart composites applications, as demonstrated by effective electro-magnetic interference shielding (Karim et al., 2021b) and de-icing applications (Karim et al., 2018). Such developments may lead to manufacturing of smart and sustainable natural fiber composites for next generation high performance engineering applications without conferring environmental problems.

Although there have been many previous reviews focusing on jute fibers and their composites (Chandekar et al., 2020; Gupta et al., 2015; Singh et al., 2018; Shah et al., 2021), there remains a lack of a review about the physical and chemical treatments of jute fiber, its modification with graphene-based materials, and their effect on the mechanical and multi-functional properties of the composites. In this review, we introduce jute fibers, their key constituents determining the mechanical properties and potential applications. We provide an overview of various surface treatments of jute fibers and their effect on mechanical properties of jute fiber reinforced composites. We then discuss graphene-based surface modifications of jute fibers, and their effect on interfacial, tensile, and multifunctional properties of fiber reinforced composites. Finally, we present our views on future research directions, and recommendations for developing next generation smart, strong, and sustainable natural fiber reinforced composites.

## Introduction to jute

### Jute plant

**Figure 1:**
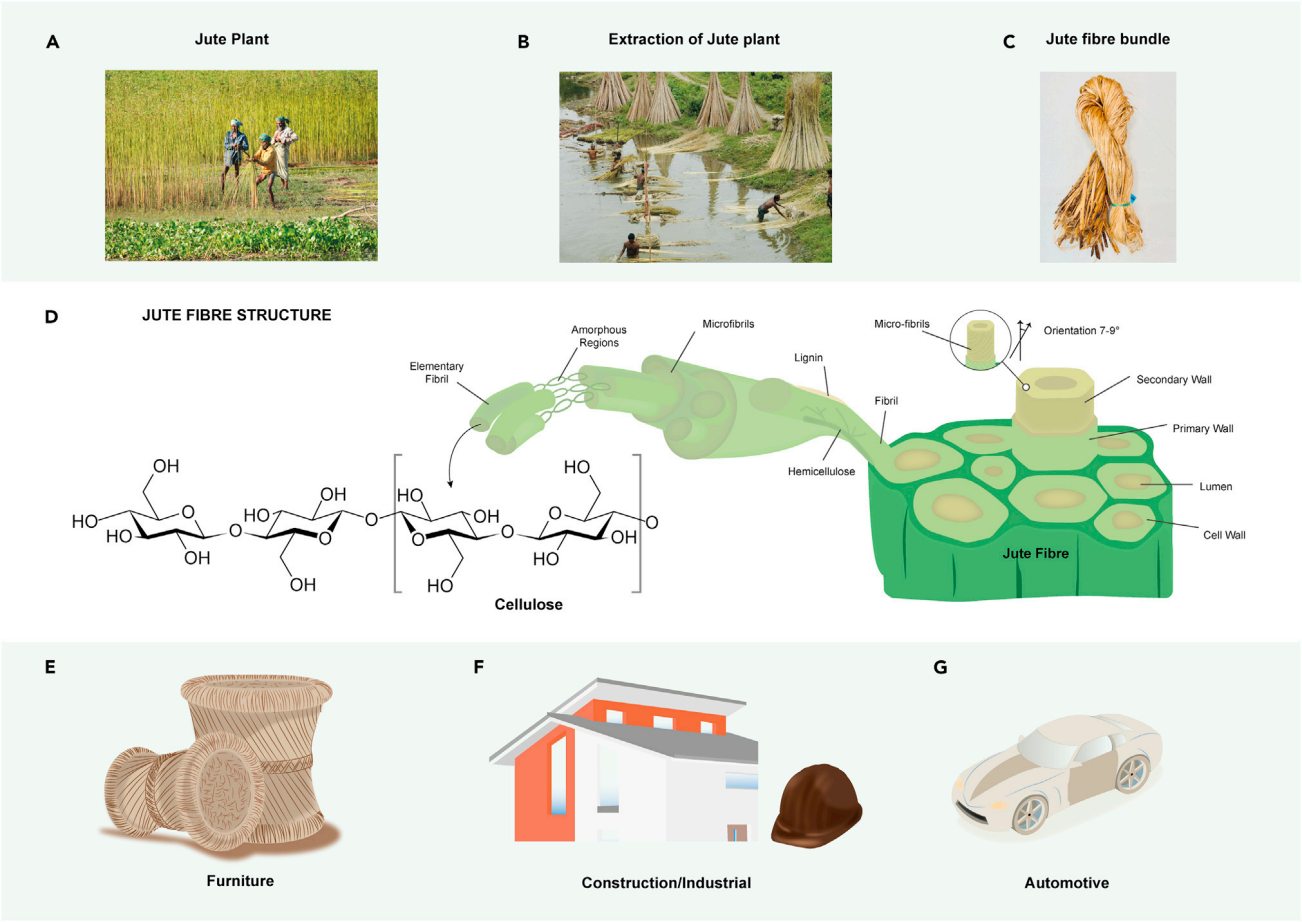
Introduction to Jute (A) Jute plant (B) the extraction of jute fibers from the plant (C) jute fibers bundle and (D) the structure of jute fiber. Applications of jute fiber and its composites: (E) furniture, (F) constructions, and (G) automobile.

Jute is a type of bast fiber, extracted from the plant, Figure 1A. Jute belongs to the Tiliaceae family with nearly 30 to 40 capsularis species (Singh et al., 2018). There are mainly two types of jute having the scientific name *Corchorus capsularis* (white jute) and *Corchorus olitorius* (Tossa jute) (Islam, 2013). For successful cultivation, jute plants need plain alluvial soil and tropical rainfall (∼125–150 mm per month), warm weather (∼20°C–40°C), and high humidity (∼70%–80%) (Rahman, 2010). The jute plant grows up from seeds to a height of 3 to 4 m, and then fibers are extracted after harvesting, which is about 4 to 5 months from the cultivation. The typical yield is ∼34 tonnes per hectare of green plants, which provides ∼2 tonnes per hectare of dry retted fiber. The current annual worldwide production of jute fiber is ∼3.2 million tonnes (Aly-Hassan, 2015). Jute is one of the world's most important natural fibers second only to cotton in terms of production. Bangladesh, India, China, Nepal, Myanmar, Thailand, and Vietnam are the major jute producing countries.

### Jute fiber

Jute fiber is extracted from the bast of the plant. The jute fiber extraction process involves retting, stripping, washing, drying, and packing. The retting process is carried out either *via* a biological method or a chemical method with the help of chemicals including ammonium oxalate, sodium sulphite, etc. However, the chemical retting process is expensive (Rahman, 2010). In the biological retting process, the jute stems are tied into a bundle, and then immersed into slow running water for ∼20 days. After the retting process, the non-fibrous materials are scraped off from the jute stem (Figure 1B) by the stripping process. After the fibers are separated, they are ready for further processing and various applications, Figure 1C. The structure of the jute fiber is illustrated Figure 1D.

The chemical composition and the configuration of the jute fiber depend on the climate conditions and degradation processes. In addition, the chemical constituents vary in different jute grades. The main chemical constituents of jute fibers are cellulose, hemicelluloses, lignin, pectin, wax, and moisture which are shown in Table 1.

**Table 1:** Chemical constituents of jute fiber

Cellulose (%)	Hemicellulose (%)	Lignin (%)	Pectin (%)	Wax (%)	Moisture (%)	References
61.2	13.2	13.7	–	0.5	–	(Mukherjee et al., 1993)
61–71	13.6-20-4	12–13	0.2	0.5	12.6	(Rowell, 2000)
61–71.5	12–13	13.6–20.4	0.2	0.5	12.6	(Goda and Cao, 2007)
71	14	13	–	–	–	(Khalil et al., 2012)
58–63	21–24	12–14	–	–	–	(Masoodi and Pillai, 2012)

The mechanical properties of jute fiber mainly depend on the nature of the plant, the cultivation environment, the locality in which it is grown, the age, and the extraction methods used (Khan et al., 2015). The tensile properties of the jute fiber are different at different positions of raw jute fiber, which become uniform throughout fibers after chemical treatments (Varma et al., 1989). The important mechanical parameters of jute fiber are presented in Table 2.

**Table 2:** Mechanical parameters of jute fiber

Tensile strength (MPa)	Young's modulus (GPa)	Elongation at break (%)	Density (g/cm^3^)	Reference
393–773	26.5	1.5–1.8	1.3	(Faruk et al., 2012)
400–800	10–30	1.5–1.8	1.46	(Saravana Bavan and Mohan Kumar, 2010)
393–773	13–26.5	1.16–1.5	1.3–1.45	(Chandrasekar et al., 2017)
393–773	19.0–26.5	1.16–1.8	1.5	(Ganesan et al., 2021)

#### Jute fiber applications

Traditionally, jute has been used to manufacture packaging materials including hessian, sacking, ropes, twines, and backing cloth for carpets. Depending on fiber properties, a wide range of diversified jute products (Figure 1E) have been produced *via* various chemical modifications and finishing processes (Islam and Xiaoying, 2016). Jute fibers have also been used as reinforcing materials for thermoplastic and thermoset composites (Shahinur et al., 2021; Mahesh et al., 2021b; Luz et al., 2015). Such fibers are sustainable, biodegradable, and available in abundance, which has drawn significant research interests over the years for fiber reinforced composites applications, because of their lower environmental impacts than synthetic fiber reinforced composites (Pougnet et al.). Therefore, the application of jute fiber composites has been increasing progressively in a wide range of areas such as aerospace, automobile, marine, sporting goods, construction, and other industries (Figures 1F and 1G). Jute fiber composites are being mostly used in the interior with very limited applications in exterior components. The hybrid jute fiber composites also have been widely used in various structural and engineering applications (Nurazzi et al., 2021). Table 3 shows the potential and in use application of jute fiber composites.

**Table 3:** Potential and in use application of jute fiber composites

Composites	Surface modification	Manufacturing technique	Main properties	Potential and in use application	Ref
Jute/epoxy	H + A + rG0 (0.5%)	VARI	TS-513 (MPa) YM-55 (GPa)	High-performance engineered application and EMI shielding device.	(Karim et al., 2021b)
Jute/epoxy	H + A + G0 (0.75%)	VARI	TS-379 (MPa) YM-45 (GPa)	Stiffness driven structural applications.	(Sarker et al., 2019)
	H + A + GnP (0.75%)		TS-294 (MPa) YM-38 (GPa)		
Jute/PP	Silane + GONP (0.05g/L)	Compounding	TS-43.8 (MPa) FS-63 (MPa)	Automotive industry, household products and so on.	(Chen et al., 2020)
Jute/epoxy	GO (0.75%)	Compression molding	TS-58 (MPa) FS-18 (MPa)	Indoor applications, it can be a good substitute for wood.	(Sadangi et al., 2021)
	FG (0.75%)		TS-59 (MPa) FS-18.8 (MPa)		
Jute/rubber/-jute/rubber/jute		Compression molding	EA- 38.7 (J) BL- 88 (m/s)	Secondary sacrificial structural applications such as claddings.	(Mahesh et al., 2021a)
Jute/polymer	Graft copolymerisation	Radiation method		Automobile industry, footwear industry, construction, home/garden furniture, and the toy sectors	(Khan et al., 2015)

H = hot water treatment, A = alkali treatment, GO = Graphene oxide coated, GnP = graphene flake coated and rGO = reduced graphene oxide coated, GF = Functionalized graphene, TS: Tensile strength, YM: Young's modulus, FS: Flexural strength, EA: Energy absorption and BL: Ballistic Limit.

## Surface modifications

The limitations of using jute fibers as reinforcements for composites are the poor mechanical properties because of the hydrophilic nature of fiber, weak fiber-matrix interfacial bonding, and poor wettability. Therefore, the surface modification of jute fibers is required. The fiber matrix interfacial bonding and moisture resistance could be enhanced by the removal of the impurities like wax, hemicellulose, lignin, and also adding other chemicals onto the fiber’s surface (Thyavihalli Girijappa et al., 2019; Chandrasekar et al., 2017). There are several jute fiber surface modification techniques which have already been used such as physical methods (Militký and Jabbar, 2015), chemical treatments (Gassan and Bledzki, 1999a), and nanomaterials modifications (Sarker et al., 2019; Karim et al., 2021b). Physical methods include stretching, calendaring, combing, cold plasma treatment, and electric discharge (Sinha and Panigrahi, 2009; Chandrasekar et al., 2017; Sever et al., 2011). Physical methods do not change the chemical composition of the fibers; however, they change the surface and structural properties of the fibers. Various chemical treatments have been used including alkalization (Brodowsky and Mäder, 2012; Gassan and Bledzki, 1999a; Roy et al., 2012; Saha et al., 2010), bleaching (Khondker et al., 2005; Alves et al., 2011), silane treatment (Wang et al., 2010; Hong et al., 2008, Dilfi et al., 2018), acetylation (Rana et al., 1997), and hot water treatment (Sarker et al., 2018). The chemical treatment of the jute fibers changes the chemical composition, as well as surface properties of the fiber.

### Alkaline treatment

Alkaline treatment is the most commonly used chemical modification technique for jute fibers. Sodium hydroxide (NaOH) is extensively used for the alkali treatment of jute fiber. The alkali treatment of jute fiber removes noncellulosic materials such as hemicellulose, lignin, wax, and oils that surround the external surface of the jute fiber. Alkali treatment of the jute fibers eliminates the moisture-related hydroxyl groups which decrease the hydrophilic nature of the fiber. When NaOH reacts with jute fiber, it produces water molecules, and Na-O- combines with the cell wall of the fiber to produce fiber-cell-O-Na groups referring to Equation 1.

(Equation 1) Fiber-cell-OH + NaOH = Fiber-cell-O-Na + H_2_O + Impurities

The alkali treatment directly affects the jute fiber properties which removes noncellulosic materials and makes the fiber more capable of rearranging themselves along the fiber direction and as well as improving the fiber matrix adhesion. This permits a better load shearing capability, which results in higher tensile strength in the composites (Wang et al., 2019b).

The NaOH treatment of the jute fiber is performed by dipping the fiber in a NaOH solution for a certain time and temperature. The efficiency of NaOH treatment of jute fiber depends on the NaOH concentration, treatment time, temperature, and material liquor ratio. A previous study (Saha et al., 2010) investigated physio-chemical properties of jute fibers treated with different concentrations (0.5%–18%) of NaOH, temperature, and time. The study showed that a 30 min dipping of the fibers in 0.5% NaOH solution followed by 30 min NaOH-stream treatment increased the tensile strength of the fiber up to ∼65%. Another study (Roy et al., 2012) suggested that a lower-concentration (∼0.5 wt% alkali treatment for a prolonged time enhanced tensile strength and elongation at break of jute fibers by ∼82% and ∼45%, respectively, and reduced the hydrophilicity by ∼50.5%.

**Figure 2:**
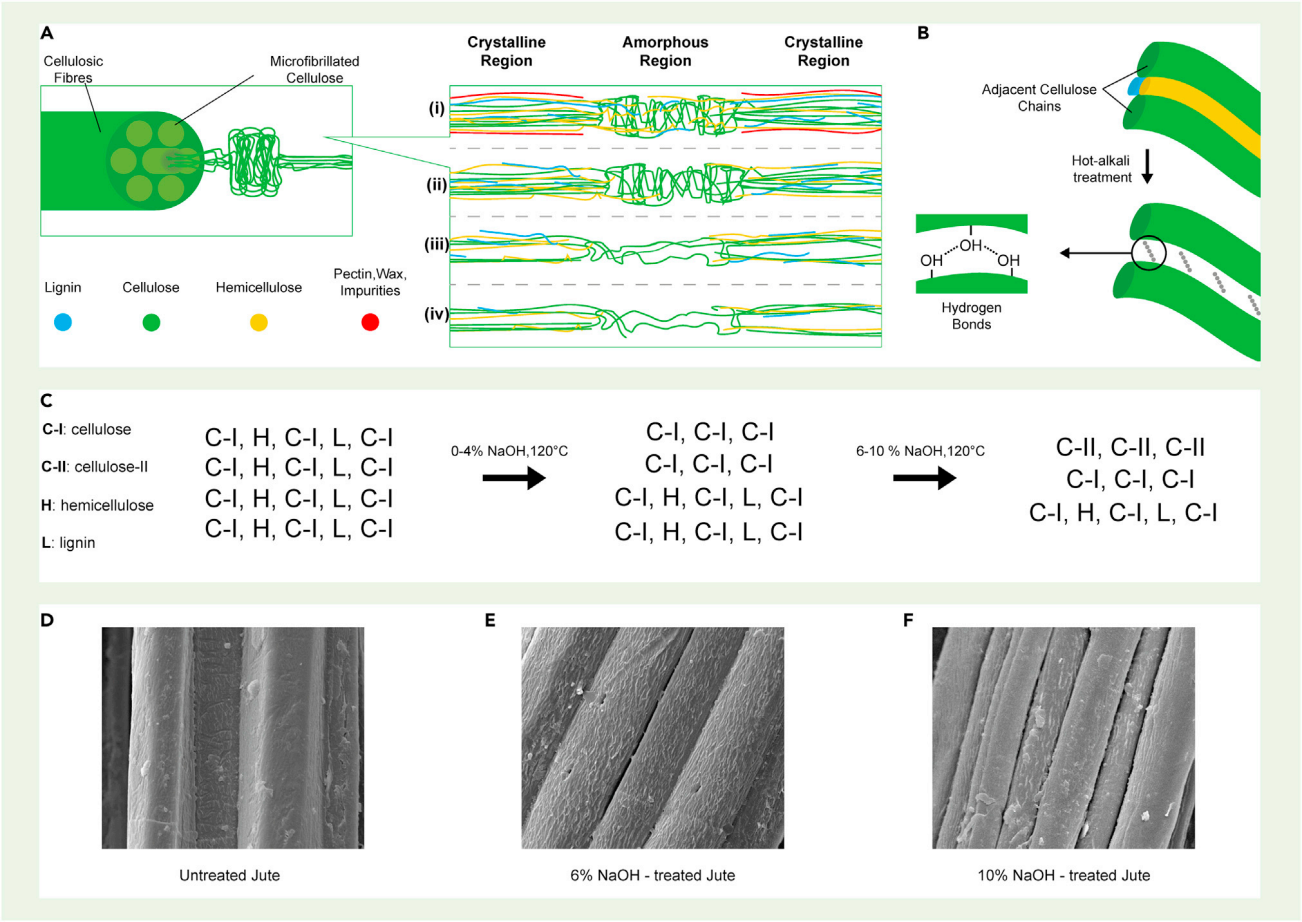
The alkali treatment of jute fiber (A) The schematic of the fine structure of cellulose and other polysaccharides of hot-alkali-treated jute fibers (i) untreated, (ii) 2% NaOH-treated, (iii) 4% NaOH-treated, (iv) 6%–10% NaOH-treated, (B) the adjacent cellulose chains, (C) the changes of cellulose, hemicellulose, and lignin contents. SEM images of jute fibers surface: (D) Untreated, (E) 6% NaOH-treated, and (F) 10% NaOH-treated. Reproduced with permission (Wang et al., 2019b).

Mechanical properties of the alkali-treated jute fiber reinforced thermoplastic and thermoset composites have been studied extensively, Table 4 (Ray et al., 2001; Gassan and Bledzki, 1999b; Sinha and Rout, 2009; Mohanty et al., 2000; Kapatel, 2021). The studies report improved tensile strength, Young's modulus, interfacial shear strength (IFSS), flexural properties, and impact strength of the composites. The effects of hot-alkali treatments with different concentrations (2%, 4%, 6%, 8%, and 10%) on the mechanical properties of the jute/epoxy composites have been investigated (Wang et al., 2019b). Composites with 6% NaOH-treated jute fabric showed the best improvement. The tensile strength, flexural strength, tensile modulus, and flexural modulus of 6% NaOH-treated fabrics reinforced composites were enhanced by 37.5%, 72.3%, 23.2%, and 72.2%, respectively, as compared with those of untreated fabrics reinforced composites. The schematic illustration of the fine structure of cellulose and other polysaccharides of hot-alkali treated jute fibers at different alkali concentrations are shown in Figures 2A–2C and SEM images of untreated alkali-treated jute fibers are also shown in Figures 2D–2F (Wang et al., 2019b). The surface morphologies of untreated jute fibers are smoothly covered with pectin, wax, and impurities, which may reduce the contact area between jute fibers and resin. The alkali treatment removes pectin, wax, and impurities, and creates many wrinkles, gaps and micro-voids. Jute fiber becomes clean and rough after alkali treatment. The crystalline structures of cellulose are improved, the spacing of adjacent cellulose chains is shortened, hydrogen bonds are formed to connect the adjacent cellulose chains, and the strength of the fiber is improved as a result of alkali treatment.

**Table 4:** Mechanical properties of untreated and alkali-treated jute fiber reinforced composites

Composites	Treatment type and time	Tensile strength (MPa	Change (%)	Flexural strength (MPa)	Change (%)	Impact strength (J)	Change (%)	Ref.
Jute/epoxy	Untreated	46.7		62.4				(Boopalan et al., 2012)
	20% NaOH 2 h	97.5	108.7	80.1	28.4			
Jute/epoxy	Untreated	25		41		2.2		(Mahesh et al., 2021b)
	5% NaOH 24 h	34	36	47	14.6	3.4	54.5	
	10% NaOH 24 h	17	−32	22	−46.3	2.55	15,9	
	15% NaOH 24 h	15	−40	18	−56.1	2.4	9.1	
Jute/epoxy	5% NaOH 24 h	12.46	–	39.08	–	2.63	–	(Gopinath et al., 2014)
	10% NaOH 24 h	10.5	–	32.5	–	2.0	–	
Jute/polyester	5% NaOH 24 h	9.24	–	44.71	–	3.25	–	
	10% NaOH 24 h	7.92	–	40.5	–	2.75	–	
Jute/epoxy	Untreated	95		98		6.35		(Kapatel, 2021)
	5% NaOH 6 h	136	43.2	140	42.9	9.1	43.3	
	10% NaOH 6 h	147	54.7	155	58.2	11.67	83.8	
	15% NaOH 6 h	156	64.2	162	65.3	15.42	142.8	
	20% NaOH 6 h	145	52.6	159	62.2	13.02	105.0

In other work, flexural properties of the 5% alkali-treated jute fiber/unsaturated-polyester resin composites for different treatment times (2 h, 4 h, and 6 h) were investigated (Sinha and Rout, 2009). Flexural strength of the composite prepared with 2 h and 4 h alkali-treated fibers were found to increase by ∼3⋅16% and ∼9⋅5%, respectively, compared to untreated jute fiber composites. Another study found that the tensile strength and flexural strength of 20% alkali-treated jute/epoxy composites improved by ∼108% and ∼28%, respectively, when compared to untreated jute/epoxy composites (Boopalan et al., 2012). Mahesh et al. studied the influence of NaOH concentrations on the treatment of jute fabric, and its effect on the mechanical properties of the jute/epoxy composites (Mahesh et al., 2021b). The jute fabric was treated with three different concentrations of NaOH (5%, 10%, and 15%) for 24 h at room temperature, and composites were manufactured using the compression molding technique. The study found that the tensile strength of the 5% NaOH treated jute/epoxy composites was enhanced by ∼36% compared to untreated jute/epoxy composite. However, the tensile strength of the 10% and 15% NaOH treated jute/epoxy composites reduces by ∼47% and ∼66.66%, respectively, compared to untreated jute/epoxy composites.

### Silane and alkali-silane treatment

**Figure 3:**
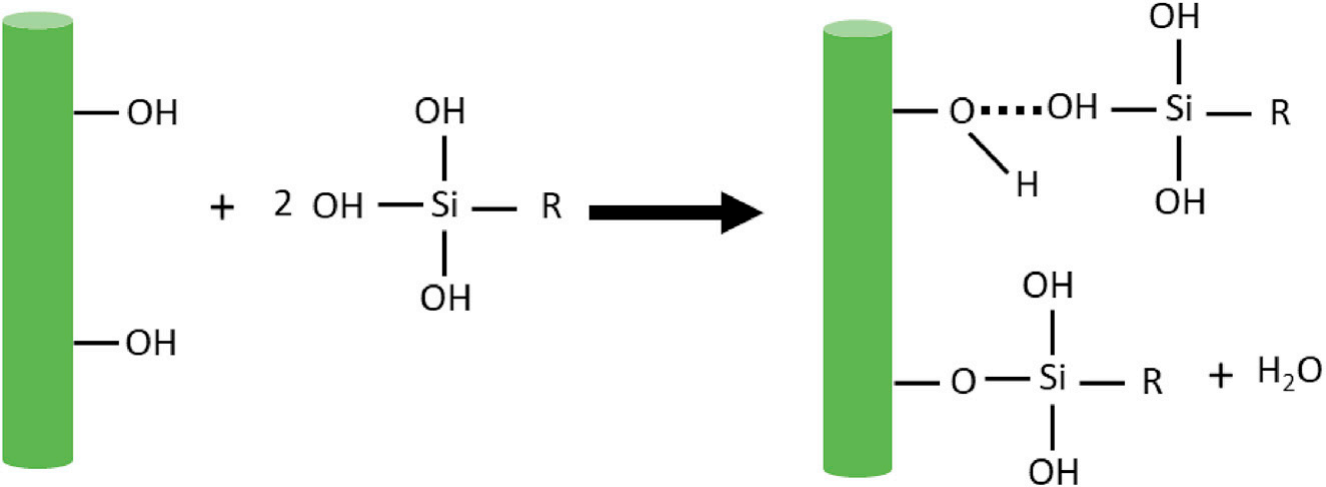
Reaction of silane with natural fiber (R representing organic group, … representing hydrogen bonding)

The silane molecules have different functional groups at both ends. One such functional group reacts with hydrophilic groups of the jute fiber and the other with hydrophobic groups in the polymer matrix to form a bridge between them (Xie et al., 2010; Pickering et al., 2016). For this reason, silane treatment improves the interfacial adhesion between jute fiber and polymer matrix. The most commonly used silanes are amino, methacryl, glycidoxy, vinyl, azide, and alkylsilanes. During the silane treatment of natural fiber (Figure 3), the hydrolysis of alkoxy groups on silane takes place to form silanol (Si–OH) groups, which can then react with hydroxyl groups on the fiber surface (Pickering et al., 2016).

In one study (Seki, 2009), the effect of oligomeric siloxane treatment of jute fabrics on mechanical properties of jute/epoxy and jute/polyester composites was studied. At first, the jute fabrics were treated with 5% (w/w) NaOH solution for 2 h, and then the alkali-treated jute fabrics were treated with 1% siloxane. The jute/epoxy and jute/polyester composites were manufactured by the hand lay-up method. The mechanical properties such as tensile, flexure, and interlaminar shear strengths of the siloxane treated composites were increased by ∼32%, ∼22%, and ∼109% for jute/epoxy composite and ∼31%, ∼37%, and ∼103% for jute/polyester composite compared to untreated jute fiber composites. It is worth mentioning that the obtained results from that study (Seki, 2009) were normalized with a 35% fiber volume fraction and presented in Table 5. In addition, the effect of amino-silane treatment on the performance of jute/polycarbonate thermoplastic composites was investigated in another study (Khan and Hassan, 2006). It was found that the tensile strength, bending strength, bending modulus, and tensile modulus of silane treated jute/polycarbonate composite were enhanced by ∼28%, ∼35%, ∼62%, and ∼70%, respectively, when compared to the untreated jute/polycarbonate composite.

**Table 5:** Mechanical properties of alkali and silane treated jute fiber reinforced composites

Composites	Treatments	Tensile strength (MPa)	Change (%)	Tensile modulus (GPa)	Change (%)	Flexural strength (MPa)	Change (%)	ILSS (MPa)	Change (%)	Ref.
Jute/epoxy	untreated	61.41		5.46		82.94		11.46		(Seki, 2009)
	5% NaOH	66.49	8.3	6.39	17	91.19	9.9	14.18	23.7	
	5% NaOH +1% siloxane	80.93	31.8	6.93	26.9	101.37	22.2	23.96	109	
Jute/polyester	untreated	45.77		3.99		59.44		8.35		
	5% NaOH	48.31	5.5	5.91	48.1	68.14	14.6	10.10	21	
	5% NaOH +1% siloxane	59.92	30.9	6.47	62.2	81.81	37.6	16.92	102.6	
Jute/epoxy	untreated					87.0		8.1		(Dilfi et al., 2018)
	1% NaOH					101.1	16.2	8.5	4.9	
	1% silane					107.8	23.9	8.9	9.9	
	1% NaOH +1% silane					128.5	47.7	9.3	14.8	
Jute/epoxy	Woven raw	56.7		7.01				51.3		(Pinto et al., 2014)
	Woven +5% NaOH+ 1% silane	55.5	−2.1	9.54	36.1			37.8	−26.3	
	UD raw	76.6		11.9				41.7		
	UD + 5% NaOH+ 1% silane	74.3	−3	13.40	12.6			38.7	−7.2	

UD = Unidirectional.

### Plasma and alkali-plasma treatment

Many researchers investigated the plasma treatment of jute fibers to manufacture the jute FRC with improved mechanical properties (Sever et al., 2011; Sinha and Panigrahi, 2009; Kafi et al., 2009). Plasma treatment introduces polar or excited groups to the fibre surface, or grafts a new polymer layer on the fiber surface that enables formation of strong covalent bonds between fiber and polymer matrix. In addition, such treatment roughens the fiber surface to enhance mechanical interlocking between fibers and polymer matrix, and improve the fiber-matrix adhesion (Yuan et al., 2002; Seki et al., 2010). The effect of plasma treatment on mechanical properties of jute fiber and jute/PLA FRC was investigated and compared with alkali-treated (AT) jute FRC (Gibeop et al., 2013). The plasma treatment of jute fibers was carried out at plasma power of 3 kV and 20 kHz for various exposure times (30s, 60s, 90s, and 120s) using helium and acrylic acid as carrier gas and monomer, respectively. The alkali treatment of jute fibers was carried out using various concentrations (3%, 5%, and 7% w/w%) of NaOH. The composites were manufactured by injection molding method using 20/80 (w/w%) jute/PLA composition. The plasma-treated jute/PLA composites provided better mechanical properties compared to untreated and alkali-treated jute/PLA composites. Tensile strength, young's modulus, and flexural strength were increased by ∼28%, ∼17%, and ∼20%, respectively, for plasma-treated jute/PLA composite, compared to untreated jute/PLA composite.

In another study (Seki et al., 2010), the effect of the low-frequency (LF) and radio-frequency (RF) oxygen plasma treatment of jute fibers on mechanical properties of jute/polyester composites was investigated. The alkali-treated jute fibers were plasma treated with LF and RF at different plasma power (30 W, 60 W, and 90 W) for 15 min. The mechanical properties of the composites were enhanced with the increase in plasma power for both LF and RF systems. The tensile strength, flexural strength, and ILSS were enhanced by ∼40%, ∼36%, and ∼72%, respectively, for LF plasma-treated jute fibers at 90 W, and by ∼63%, ∼77%, and ∼129%, respectively, for RF plasma-treated fibres at 90 W, when compared to those of untreated jute fibres.

### Alkali-bleaching

Alkali treatment followed by hydrogen peroxide (H_2_O_2_) bleaching was carried out to modify short jute fibers, and the effect of such treatment on composites’ tensile properties was investigated (Rajesh and Prasad, 2014). The jute fibers were treated with various concentrations of NaOH (5%, 10%, and 15%) followed by H_2_O_2_ (10 mL/L) treatment. The composites were manufactured with different weight% (5%, 10%, 15%, 20%, and 25%) of treated and untreated jute fiber content with PLA matrix *via* an injection molding technique. Jute/PLA composites with 10% NaOH treated fibers followed by H_2_O_2_ bleaching at 20% fiber loading provided ∼7.5% and ∼40% higher tensile strength and modulus, respectively, than those of untreated jute fiber/PLA composites.

## Graphene-based high-performance jute fiber composites

Graphene-based surface modifications for jute fiber composites can be carried out either *via* treating reinforcing materials (e.g., fibers) with graphene and its derivatives (Sarker et al., 2019) or mixing such materials with a suitable polymer matrix (Sadangi et al., 2021; Costa et al., 2020). A wide range of coating processes has been used to deposit graphene-based materials on the fiber or fabric surface. Among them, dip-coating (Sarker et al., 2019, Dilfi et al., 2019) and spray coating (Wang et al., 2019a) are the most popular techniques for coating natural fiber or fabric. Dip coating is the easiest and effective method where the fibre is immersed into the dispersion of graphene and its derivatives. In the spray coating process, simple spraying of graphene-based materials can be carried out directly on the fiber surface to obtain a homogenous surface covering of deposited materials. In the matrix modification process, the graphene-based materials are mixed with a suitable resin and then infused them the fiber reinforced materials using existing resin impregnation system.

### Graphene-based materials

The isolation of graphene, a one-atom-thick two-dimensional layer of sp^2^-bonded carbon (Georgakilas et al., 2015; Karim et al., 2019), has unveiled a wide range of novel two-dimensional materials with outstanding physicochemical properties (Novoselov et al., 2012; Park and Park, 2021) and is therefore highly admired by the researcher community for both blue sky and application-based research (Afroj et al., 2021a, 2021b; Pereira et al., 2020b). A possible route of harnessing these properties for applications might be the incorporation of graphene into composite materials (Pereira et al., 2020a; Ahmed et al., 2021). The fundamental requirements for such applications include high scalability, reliability, and cost-effective production process. The final properties of the graphene-based material depend on the number of graphene layers, average lateral dimension, atomic carbon/oxygen ratio, surface chemistry, surface area, and material purity (Wick et al., 2014, Da Luz et al., 2020). The poor solubility of graphene limits its wide -scale industrial applications (Johnson et al., 2015). Among the two oxidative derivatives of graphene, graphene oxide (GO) exhibits less electrical conductivity and high solubility in water (Karim et al., 2021a), and reduced graphene oxide (rGO) exhibiting properties between graphene and GO (Mohan et al., 2015), is sparingly dispersible in water or other solvents and offers admirable electrical conductivity (Yusuf et al., 2019).

There are various synthetic approaches for the preparation of graphene and its derivatives, which could be categorized under two groups-namely bottom-up and top-down techniques; all producing graphene with different morphologies, different flake diameter and thickness, corrugation and surface chemistry (Lee et al., 2019; Madurani et al., 2020). The bottom-up technique, usually employed for small scale production, includes methods such as micromechanical peeling of graphite, chemical vapor deposition (CVD), plasma enhanced CVD, and epitaxial growth of graphene on SiC substrate. In these techniques, graphene flakes are deposited on a suitable substrate from carbon sources and allow the production of contamination-free graphene with control over initiation and growth of graphene by correct choice of substrate (Liu et al., 2020). However, bottom-up methods are not very popular for application-based research work and large-scale production due to their low yield, complex processing and associated higher cost (Yang and Wang, 2016). On the other hand, for larger scale production of graphene, several top-down techniques are followed where graphene is mainly exfoliated from bulk (Luo et al., 2012). Benefits of such processes include solution based processability, ease of implementation, and higher yield compared to bottom-up processes (Yang and Wang, 2016). Liquid phase exfoliation, electrochemical exfoliation using ionic intercalation, chemical oxidation of graphite to make GO and sometimes followed by the reduction to make rGO, have been employed as popular top-down techniques (Narayan and Kim, 2015).

Among several exfoliation methods, the liquid phase exfoliation (LPE) is a versatile, scalable, sustainable, and cost-effective method to produce single-layer graphene, and therefore used widely (Ren et al., 2018; Bonaccorso et al., 2016). Basically, in liquid phase exfoliation (LPE) processes, ultrasonic or shear energy are applied to break inter-sheet forces of carbon in presence of a stabilizing liquid (Hernandez et al., 2008), either in a non-aqueous solution (Hernandez et al., 2008; Hassoun et al., 2014; Khan et al., 2010) or an aqueous solution with surfactant (Hernandez et al., 2008). Possibly because of this in-plane fracture during the exfoliation and purification process to separate unexfoliated flakes, the flake size of graphene produced by LPE is mostly below 1 μm^2^ (Lotya et al., 2009; Hernandez et al., 2008; Khan et al., 2010; Maragó et al., 2010). Among other LPE processes, microfluidization produces graphene with 100% yield by weight (Y_w_) and a higher concentration compared to other LPE processes as the force acts over the whole volume of the liquid, whereas , bath sonication, tip sonication, and shear exfoliation generally produce graphene of lower concentration as well as lower yield (up to 2%) by weight (Y_w_). Electrochemical expansion process offers much better yield by weight (Y_W_ >70%). To enjoy the distinctive properties of graphene it is important to produce and maintain graphene as individual sheets. However, graphene sheets tend to agglomerate and even restack to form graphite through van der Waals interactions (Li et al., 2008). Therefore, the other viable way to produce solution-processable graphene on a large scale is through reduction of graphene oxide with added functionality (Liu et al., 2012b; Lonkar et al., 2015). Various types of chemical reduction (*via* ascorbic acid, sodium hydrosulphite, hydrazine hydrate, hydriodic acid with acetic acid, etc), thermal reduction and electrochemical reduction can be used to achieve different properties in rGO depending on various final applications (Karim et al., 2017, 2021a; Afroj et al., 2021a, 2021b). However, the disadvantage of this route is that none of these reduction processes can completely reduce and eliminate the many structural defects introduced by the oxidation process (Zhang et al., 2010; Kang et al., 2009; Yang et al., 2009). The proper fine tuning of the synthesis protocols still remains a challenge (Li et al., 2020) for obtaining quality graphene-based materials for their wide range of applications (Yusuf et al., 2019; Ren et al., 2018).

Lower crystallinity, hydrophilicity, and inherent electrical insulation properties of jute fiber results in lower mechanical, poor interfacial, and electrical properties of jute fiber reinforced composites that limits their application as multifunctional composites (Gassan and Bledzki, 1999b; Karim et al., 2021b). Few carbon based materials were reported to enhance properties of jute fiber composites earlier (Zhuang et al., 2011; Islam et al., 2020; Saiteja et al., 2020; Tzounis et al., 2014); however, a recent study reported for the first time the incorporation of graphene and its derivatives to design high-performance natural fiber composites (Sarker et al., 2018). In this study alkali-treated jute fibers were coated with graphene flakes and GO which enhanced interfacial shear strength by ∼236% and tensile strength by ∼96%. Considering the improved characteristics of rGO making it an ideal material for composite, Karim et al. incorporated rGO with jute fiber which significantly improved the tensile strength by ≈ 183% along with the Young's modulus of the composites by ≈450% (Karim et al., 2021b).

### Graphene-based jute: Single fiber properties

**Figure 4:**
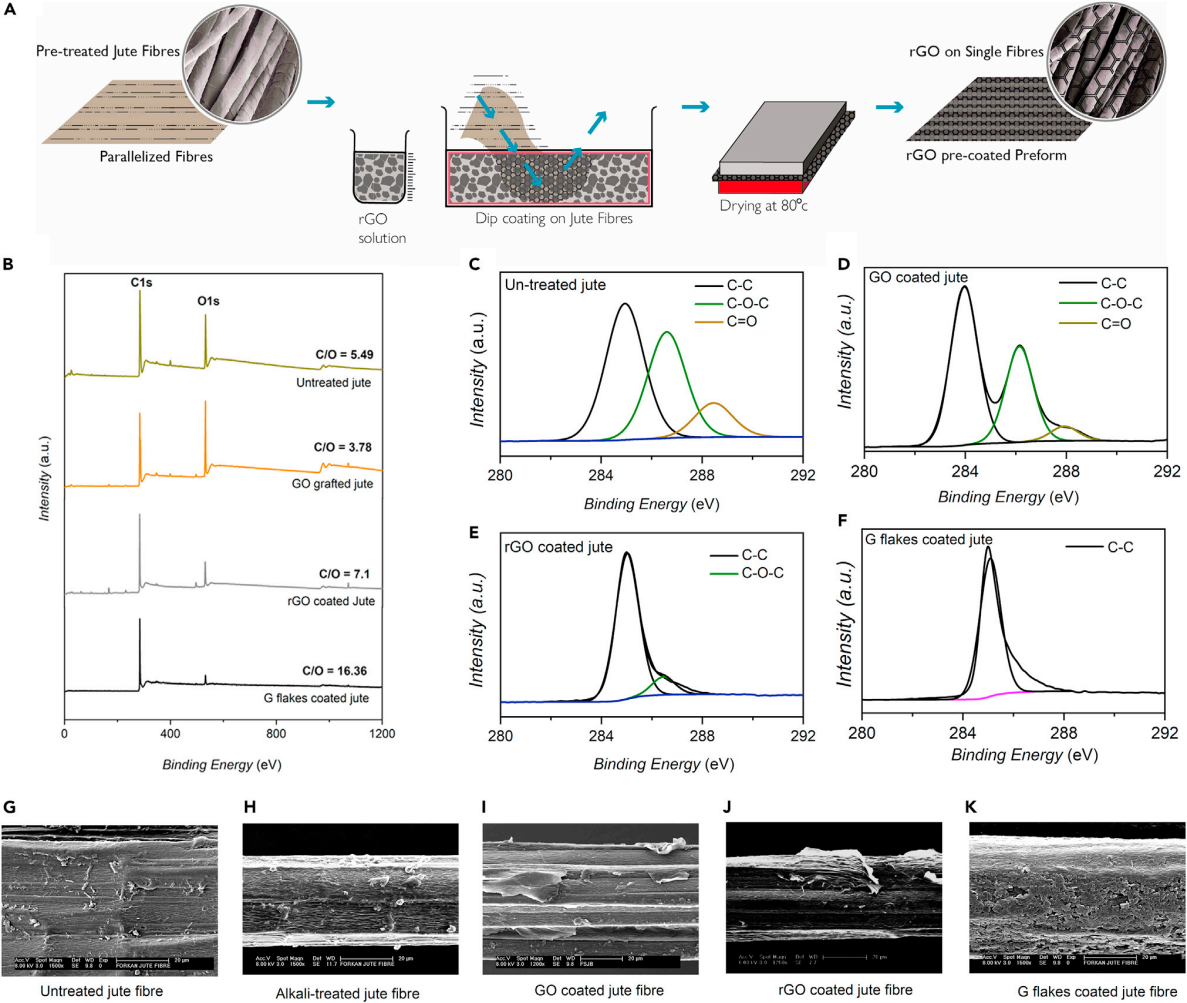
Graphene-based jute fibers: Coating, surface functionalities, and morphologies (A) Schematic diagram showing 2D material coating process on jute fibers and the preparation of 2d material-coated jute fiber preforms, (B) wide scan XPS spectrum of untreated, GO, rGO, and G flakes coated jute fiber (C) high resolution C(1s) XPS spectrum of untreated jute fiber, (D) high resolution C(1s) XPS spectrum of GO-coated jute fiber, (E) high resolution C(1s) XPS spectrum of rGO-coated jute fiber, (F) high resolution C(1s) XPS spectrum of G flake-coated jute fiber. SEM image of (G) untreated jute fiber (X1500); (H) HA0.5 treated jute fiber (X1500); (I) GO treated jute fiber (X1200); (J) rGO treated jute fiber e (X1250); and (K) G flakes treated jute fiber (X1500). Reproduced with permission (Sarker et al., 2018, Karim et al., 2021b).

The surface modification of jute fibers with graphene oxide (GO) (Sarker et al., 2018), reduced graphene oxide (rGO) (Karim et al., 2021b), and graphene flakes (G) (Sarker et al., 2018, 2019) have been carried out. Jute fibers were chemically treated with hot water and 0.5% NaOH to remove noncellulosic materials. The chemically treated jute fibers were then coated with graphene derivatives *via* simple dip-coating method, Figure 4A. The jute fibers were immersed in GO, rGO, and G dispersions for 30 min and subsequently dried at 80°C for 30 min. The surface characteristics and mechanical properties of untreated and treated jute fibers were investigated.

The wide-scan X-ray photoelectron spectroscopy (XPS) spectra in Figure 4B show that the C/O ratio of jute fibers decreased from ∼5.5 to ∼3.8 after coating with GO, because of the presence of oxygen-containing functional groups in GO (Abdelkader et al., 2017). The C/O ratio of rGO coated jute fiber increased to ∼7.1 because of the partial restoration of graphene structures. The maximum C/O ratio of ∼16.4 was obtained for jute fibers coated with G flakes, which may be because of the absence of oxygen-containing functional groups in their structures (Afroj et al., 2020). The high resolution C1s XPS spectra of untreated and GO coated jute fibers are similar, and show the presence of three main components: C-C bond (≈284.5 eV) in cellulosic structure, C-O-C groups (hydroxyl and epoxy, ≈286.5 eV), and C=O groups (carbonyl, ≈288.3 eV), Figures 4C and D. The peaks associated with the oxygen functional groups significantly diminished after coating with rGO, with small amounts of residual oxygen functional groups left as evident from the peak around 287.5 eV, Figure 4E. The C1s spectrum of G-coated jute fibers is similar to graphene or graphite, mainly dominated by C–C/C=C, Figure 4F. Therefore, G-flakes are loosely attached to cellulosic jute fibers, because of the absence of oxygen-containing functional groups (Sarker et al., 2018).

Scanning electron microscope (SEM) images show smooth untreated jute fiber surface (Figure 4G), because of the presence of wax, fat, lignin, and hemicellulose, which becomes rough after alkali treatment (Figure 4H) because of the removal of the noncellulosic materials. It was found that the GO and rGO uniformly coated the jute fiber surface (Figures 4I and 4J) (Sarker et al., 2018; Karim et al., 2021b), because of the chemical interaction between the hydroxyl group of the jute fiber and the oxygen functional group of the GO and rGO. However, the G-flakes provided a uniform coating with plenty of unfixed graphene flakes on the fiber surface (Figure 4K) (Sarker et al., 2018).

**Figure 5:**
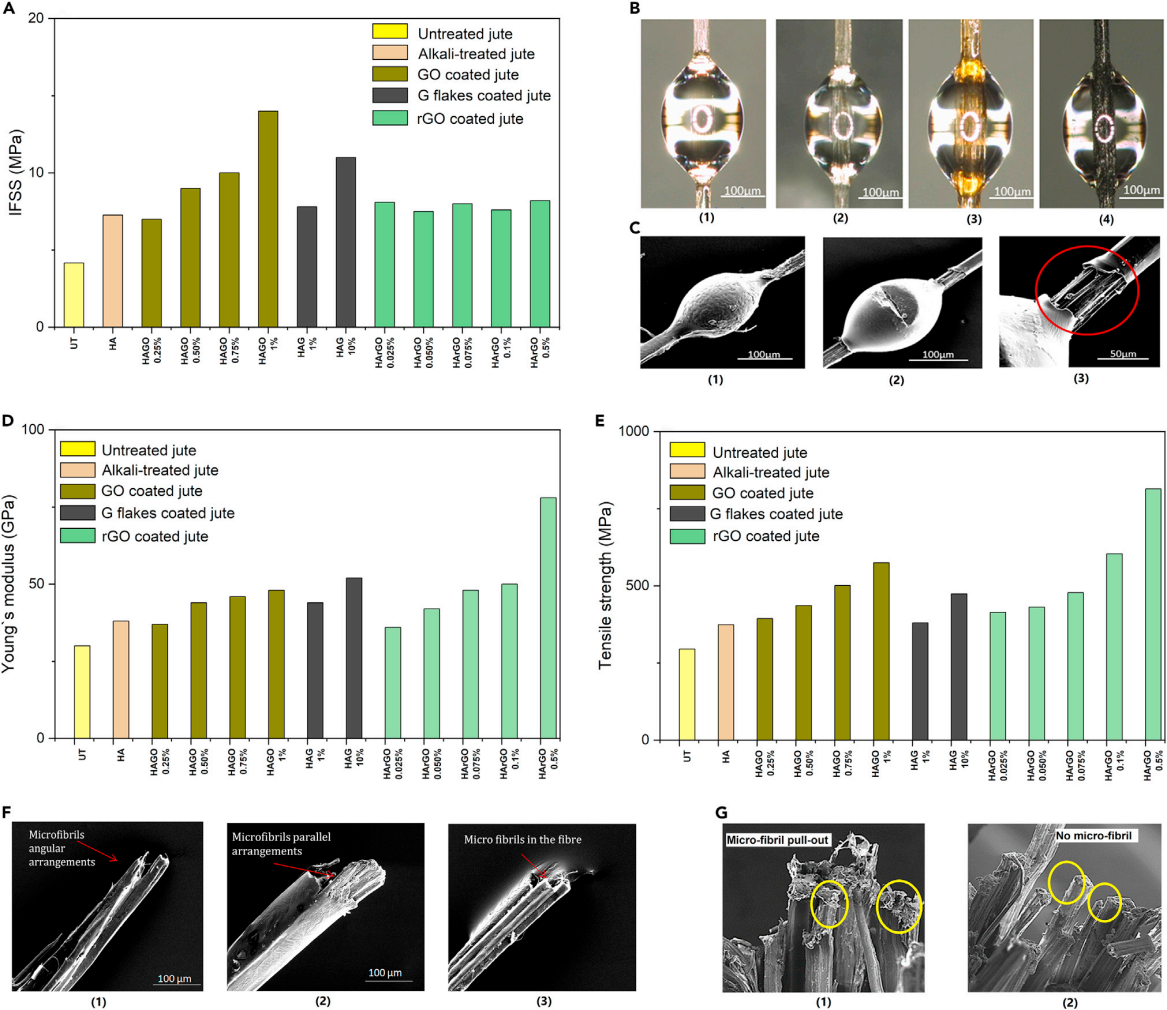
High performance graphene-based jute fibers (A) Interfacial shear strength (IFSS) of untreated, alkali-treated, GO, rGO, and G flake-coated jute fibers, (B) Optical microscopic images of the microdroplet of epoxy on (1) untreated; (2) alkali-treated; (3) GO-coated; and (4) G flake-coated jute fibers (X200) before microbond test, (C) SEM image of microdroplets of epoxy on jute fiber (1) before microbond test; (2) after microbond test; and (3) de-bonded area (red circle line) after microbond test (X250), (D) Young’s modulus and (E) tensile strength of untreated, alkali-treated, GO, rGO, and G flake-coated jute fibers, (F) SEM images of the fracture specimen after single fiber tensile test (1) untreated; (2) GO-coated; and (3) G flake-coated jute fiber (X250) and (G) SEM images of the fracture specimen after single fiber tensile test (1) untreated and (2) rGO-coated jute fiber (X250). Reproduced with permission (Sarker et al., 2018, Karim et al., 2021b).

Tensile properties and interfacial shear strength (ILSS) of the untreated, alkali-treated, GO, rGO, and G-coated jute fibers were also investigated (Sarker et al., 2018; Karim et al., 2021b). A single fiber microbond pull-out test was used to measure ILSS. Optical and SEM images of microdroplets of epoxy on jute fiber before and after microbond test are shown in Figures 5B and 5C and IFSS results are shown in Figure 5A. The ILSS of GO (1%), rGO (0.5%), and GnP (10%) coated single jute fiber composites was increased by ∼236%, ∼97%, and ∼164%, respectively compared to untreated jute fiber epoxy composites. Such a significant improvement of IFSS with GO is associated with the presence of a huge amount of oxygen functional group such as hydroxyl (-OH), epoxide (C-O-C), carbonyl (C=O), and carboxyl (O–C=O) in GO. Such functional groups interact with the groups of epoxy resin and form a strong mechanical interlocking at the fiber/matrix interface *via* suitable bonding. Similarly, there was a large increment in IFSS of G-flakes coated jute fibers (Sarker et al., 2018), which may be related to the strong mechanical interlocking of G-flakes onto the rough and porous jute fiber surface. However, IFSS of rGO coated jute fibers increased slightly. Nevertheless, the effect of rGO coating on improving IFSS value is better than other natural and synthetic fibers modified by traditional alkali and nanomaterials (Karim et al., 2021b).

The single fiber tensile test of untreated and treated jute fibers was carried out *via* mounting the fiber on the paper frame. The single fiber tensile test shows the increase of tensile strength from 295 MPa to 575, 814, and 474 MPa, and Young's modulus from 30 GPa to 48, 78, and 52 GPa for 1% GO, 0.5% rGO, and 10% G flakes coated jute fibers (Karim et al., 2021b; Sarker et al., 2018), Figures 5D and 5E and Table 6. Such significant improvement in tensile properties is attributed to the strong bonds between the functional groups of graphene derivatives and those of chemically treated jute fibers. In addition, the coating with G flakes provides increased stiffness of jute fiber, which enables the removal of stress concentrations on the fiber surface during tensile loading, resulting in the enhancement of the tensile properties. The SEM images of the fractured specimen after the tensile test show uneven microfibrils fracture for untreated jute fibers because of stress concentration between the cellulosic microfibrils in the fiber, Figures 5F1 and 5G1. However, a linear breakage of microfibrils was observed with graphene derivatives coated jute fiber (Figures 5F2, 5F3, and 5G2) which is responsible for more loading capacity along with the tensile deformation. The rGO coating on jute fibers provides the highest tensile properties obtained so far with alkali treatment and nanosurface engineering of jute fibers (Karim et al., 2021b; Sarker et al., 2018).

**Table 6:** Tensile properties of untreated, alkali-treated, GO, rGO, and G flakes coated jute fibers

Fiber	Treatment	Tensile strength (MPa)	Change %	Young's modulus (GPa)	Change %	Ref
Jute	Untreated	295		30		(Sarker et al., 2018)
	H	293	−1	29	−3	
	H + A	374	27	38	27	
	H + A + GO 0.25%	394	34	37	23	
	H + A + GO 0.50%	436	48	44	47	
	H + A + GO 0.75%	501	70	46	53	
	H + A + GO 1%	575	95	48	60	
	H + A + G 1%	380	29	44	47	
	H + A + G 10%	474	61	52	73	
	H + A + rGO 0.025%	414	40	36	20	(Karim et al., 2021b)
	H + A + rGO 0.050%	431	46	42	40	
	H + A + rGO 0.075%	478	62	48	60	
	H + A + rGO 0.1%	604	105	50	67	
	H + A + rGO 0.5%	814	176	78	160	

H = Hot water treatment, A = Alkali treatment, GO = Graphene oxide coated, G = Graphene flake coated and rGO = Reduced graphene oxide coated.

### Ultrahigh performance of graphene-based jute fiber composites

**Figure 6:**
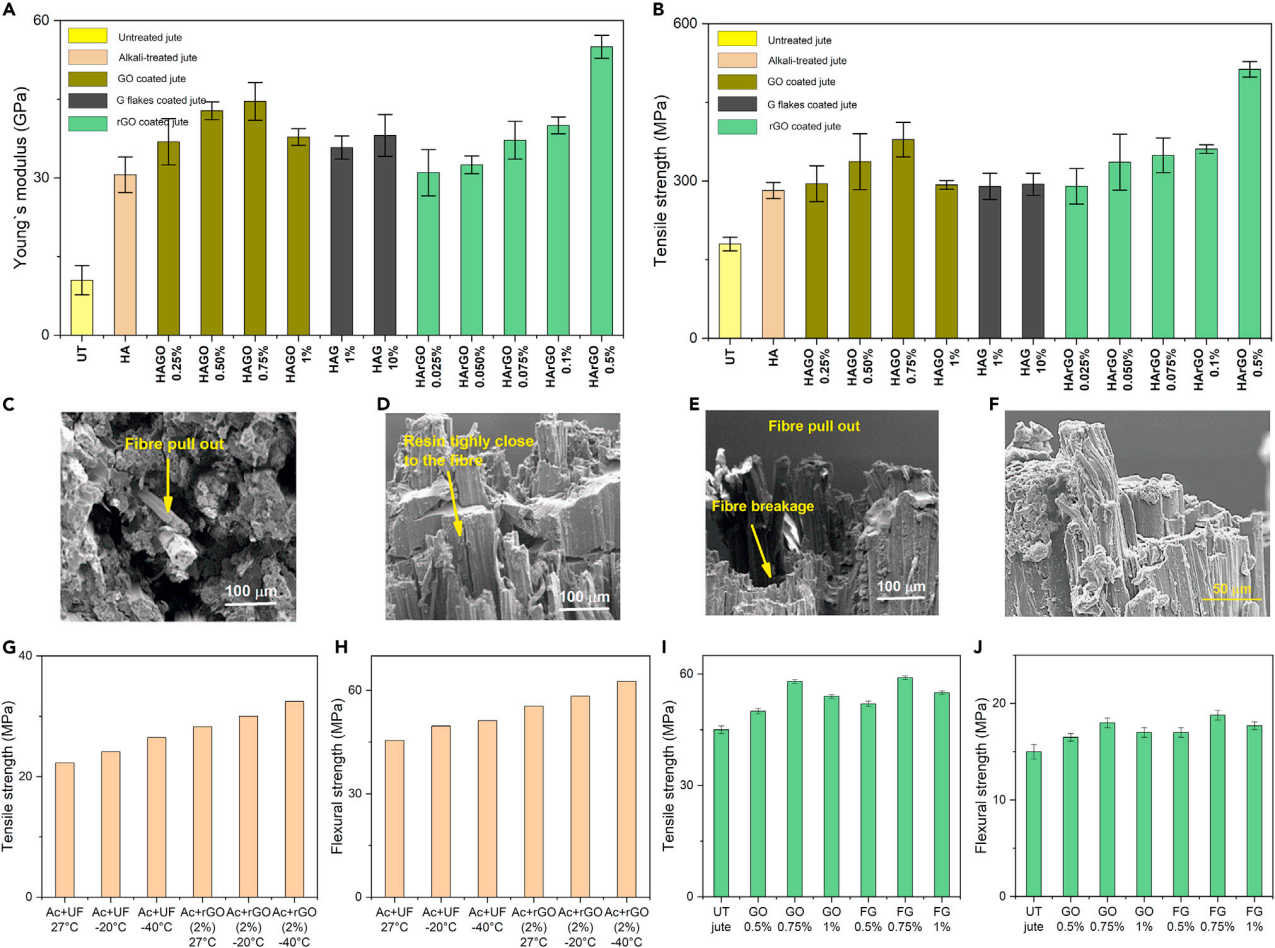
Ultrahigh performance of graphene-based jute fiber composites Longitudinal (A) Young’s modulus, (B) tensile strength of untreated, alkali-treated and graphene materials treated jute fiber/epoxy composites. SEM images of the fracture surfaces of (C) untreated, (D) GO coated, (E) graphene-coated, and (F) rGO coated jute/epoxy composites after the longitudinal tensile test. Reproduced with permission (Sarker et al., 2018, Karim et al., 2021b). (G) Tensile strength (H) flexural strength of acetone treated unfilled and rGO filled jute/epoxy composites at different temperatures.Reproduced with permission (Pa and M, 2019).(I) Tensile strength and (J) flexural strength of untreated, GO and FG based jute/epoxy composite. Reproduced with permission (Sadangi et al., 2021).

Although mechanical properties of individual fibers have been increased significantly *via* treating jute fibers with graphene-based materials, the main challenge is how such excellent properties can be translated to jute FRC for real world applications. In our previous study (Sarker et al., 2019), we addressed this challenge *via* a novel strategy of combining physical and chemical modifications of jute fiber preforms to manufacture next generation jute FRC. Before graphene coating, the jute fiber mats were prepared by physical and chemical treatment of the jute fibers. Unidirectional (UD) jute/epoxy composites were prepared with alkali-treated and graphene materials-coated jute fibers *via* vacuum-assisted resin infusion (VARI) method. The longitudinal tensile strength, Young’s modulus, and strain% of jute/epoxy composite were increased with the increase of concentration of GO up to 0.75mg/mL because of the nano-engineering effect of graphene materials on jute fibre but after this concentration of GO deteriorate the tensile properties of composites because of the agglomeration of GO flakes, Figures 6A and 6B and Table 7. In addition, mechanical properties of rGO-based natural jute fibres UD composites were investigated (Karim et al., 2021b). The Young's modulus, tensile strength, and tensile strain of the rGO-based jute FRC increased with the increase in rGO concentrations, and the maximum improvement was achieved with 0.5% rGO coated jute epoxy composites, Figures 6A and 6B. The combination of all physical and chemical treatments together with rGO coating resulted in ≈450% and ≈183% improvement in Young's modulus and the tensile strength of the composites, which is the highest improvement in the tensile properties of any kind of natural fiber composites reported in the literature. SEM images of the fracture surfaces of the graphene material-based jute/epoxy composites are shown in Figures 6C–6F.

**Table 7:** Mechanical properties of untreated, hot water, alkali, graphene flakes, graphene oxides, and reduced graphene oxide treated jute fibre/epoxy composites

Composites	Treatment/modification	V_f_	Tensile strength (MPa)	Young's modulus (GPa)	Strain to failure (%)	Flexural strength (MPa)	ILSS (MPa)	Ref
Jute/epoxy	Untreated	0.24	180 ± 13	10.5 ± 2.8	0.80 ± 0.05			(Sarker et al., 2019)
	H	0.50	230 ± 19.7	26.5 ± 3.45	0.85 ± 0.09			
	HA (0.5%)	0.54	282 ± 15.4	30.6 ± 3.4	0.86 ± 0.09			
	HA + GO (0.25%)	0.54	295 ± 33.9	36.9 ± 4.4	0.84 ± 0.07			
	HA + GO (0.50%	0.54	337 ± 53.3	42.8 ± 1.7	0.94 ± 0.10			
	HA + GO (0.75%)	0.56	379 ± 33	44.6 ± 3.6	0.93 ± 0.05			
	HA + GO (1%)	0.56	292.7 ± 8.4	37.8 ± 1.6	0.78 ± 0.01			
	HA + GnP(1%)	0.55	290 ± 25	35.8 ± 2.2	0.84 ± 0.08			
	HA + GnP(10%)	0.55	294 ± 21	38.1 ± 4.0	0.80 ± 0.07			
Jute/epoxy	HA + rGO (0.025%)	0.54	290 ± 33.9	31 ± 4.4	0.84 ± 0.07			(Karim et al., 2021b)
	HA + rGO (0.050%	0.54	336 ± 53.3	32.5 ± 1.7	0.86 ± 0.10			
	HA + rGO (0.075%)	0.56	349 ± 33	37.2 ± 3.6	0.93 ± 0.05			
	HA + rGO (0.1%)	0.56	361 ± 8.4	40 ± 1.6	0.99 ± 0.01			
	HA + rGO (0.5%)	0.60	513 ± 15	55 ± 2.2	1.13 ± 0.08			
Jute/Epoxy	UF 27°C	0.39	22.26			45.45	2.22	(Pa and M, 2019)
	UF -20°C		24.12			49.65	4.11	
	UF -40°C		26.46			51.21	5.40	
	Ac + rGO (2%) 27°C		28.26			55.41	2.43	
	Ac + rGO (2%) −20°C		30.01			58.31	4.34	
	Ac + rGO (2%) −40°C		32.44			62.64	5.70	
Jute/Epoxy	Pure jute	60%	45 ± 1.06			15 ± 0.75		(Sadangi et al., 2021)
	GO (0.5%)		50 ± 0.75			16.5 ± 0.4		
	GO (0.75%)		58 ± 0.50			18 ± 0.50		
	GO (1%)		54 ± 0.50			17 ± 0.50		
	FG (0.5%)		52 ± 0.75			17 ± 0.50		
	FG (0.75%)		59 ± 0.50			18.8 ± 0.50		
	FG (1%)		55 ± 0.50			17.7 ± 0.40		

(UF = Unfilled, Ac = Acetone treated, FG = Functionalized graphene).

In another study, mechanical and thermal properties of the graphene oxide nanoplatelets (GONPs) modified jute/polypropylene composites were investigated and compared with untreated jute/PP composites (Chen et al., 2020). Firstly, the jute fiber surface was treated with a silane coupling agent and then the jute fiber was coated with GONPs. Three different types of silane coupling agents 3-aminopropyltriethoxysilane (KH550, 3-Glycidoxypropyltrimethoxysilane (KH560) and 3-Methacryloxypropyltrimethoxysilane (KH 570) were used. Among them, KH 570 provided the best performance. The GOPNs effectively improved the fiber matrix interfacial adhesion. The study revealed that the tensile and flexural strength of the silane and GONPs modified jute/PP composites increased by 16.2% and 12.4%, respectively, compared to untreated jute/PP composites. The crystallization temperature of silane and GONPs modified jute fiber PP composites also increased by ∼3°C.

The mechanical behavior of rGO filled jute/epoxy composites was investigated at different temperatures to quantify the effect of temperature and rGO filler on the mechanical properties of the composites (Pa and M, 2019). The tensile strength, compressive strength, flexural strength, energy absorption, and ILSS were analyzed at various temperature conditions with 2% rGO-based jute/epoxy composites and compared with unfilled jute epoxy composites. The rGO filled jute/epoxy composite exhibited better mechanical properties at all temperatures compared to the unfilled jute/epoxy composites Figures 6G and 6H and Table 7. The hybrid composites tested for strength at sub-zero temperatures showed enhanced mechanical properties compared to room temperature conditions.

The influence of GONPs and silica-decorated graphene oxide (SiO_2_@GONPs) at different loadings (0, 0.1, 0.3, and 0.5 wt.-% with respect to the matrix) on the flexural and high-velocity impact properties of jute fiber/epoxy composites was also investigated (Amirabadi-Zadeh et al., 2021). The multiscale composites (jute/epoxy, GONPs/jute/epoxy, and SiO_2_@GONPs/jute/epoxy) were prepared using the static pressing assisted hand layup method. The most promising results were obtained with 0.3 wt.-% of SiO_2_@GONPs modified jute/epoxy composites. The study found that the flexural strength, energy absorption capability, and impact limit velocity of the 0.3 wt.-% SiO_2_@GONPs modified jute/epoxy composite improved by ∼40%, ∼61%, and ∼28%, respectively, from those of the neat jute/epoxy composites. In other study, the effect of two different nanofillers such as graphene oxide (GO) and functionalized graphene (FG) on the mechanical properties of the Jute/epoxy composites have been investigated at different nanofiller contents (0.5, 0.75, and 1 wt.-%) (Sadangi et al., 2021). It was found that jute/epoxy composites with nano fillers have better mechanical properties such as tensile and flexural strength compared to composite without any nanofiller, and composites with 0.75 wt.-% nanofiller exhibited best results. Jute/epoxy composites with FG exhibited better mechanical properties than the jute/epoxy composites with GO, Figures 6I and 6J.

Drilling and milling processes are widely used during the assembly of composite parts with other components (Liu et al., 2012a). The surface finish attained after drilling and milling operation is an issue of concern as a rough surface may lead to crack initiation and finally failure of components. The milling and drilling performance of graphene-modified jute/basalt hybrid composites have been investigated and compared with pure jute and pure basalt fiber composites (Kishore et al., 2021). The addition of graphene (0.2 wt.-%, 0.4 wt.-% and 0.6 wt.-%) to hybrid composites improved lubrication and led to reduced surface roughness.

## Conclusion and outlook

With growing environmental concerns with petrochemical-based synthetic fibers (Uddin et al.,2021; Karim et al., 2020), researchers have turned their attention to biodegradable fibers from renewable sources. However, such materials suffer from poor performance properties and thermal stability (Karim et al., 2014). Therefore, key challenge is to produce sustainable, biodegradable, and lightweight composites which provide combinations of excellent strength, stiffness, toughness, and multi-functionalities. An overview of jute surface modifications using the physical and chemical methods and further modification with graphene and its derivatives is provided. In addition, the effect of such surface treatments and graphene-based modifications on mechanical and multifunctional properties of jute FRC is extensively reviewed. A great effort has been paid to improve the mechanical and functional properties of jute FRC *via* physical and chemical treatments of jute fibers, and also incorporating graphene-based 2D materials. A wide range of studies has been carried out to improve the interfacial adhesion between jute fibers and polymer matrix such as physical (plasma), chemical (alkali, bleaching, silane), or combined physical and chemical (alkali-plasma) or different chemical (alkali-bleaching, alkali-silane) treatments. Such treatments have improved the mechanical and interfacial properties of the composites. However, this improvement is not sufficient to use developed composites as an alternative to synthetic FRC. A combined physical and chemical treatment using graphene-based materials modified jute fiber/epoxy composites showed a significant increase in the tensile and interfacial properties and comparable specific properties to those of glass fibers with added multifunctionalities. There has been very limited research that used graphene-based materials to modify jute fiber and manufacture FRC. Therefore, more research is needed for better understanding of the interactions among graphene-based materials, reinforcing fibers and matrix materials to promote this novel class of composites materials for industrial applications.

There is a growing interest in sustainable, biodegradable, and lightweight smart materials for structural composites applications, that would offer unprecedented combinations of stiffness, strength, toughness and multifunctionality. Most of these researches have used petrochemical-based or nonbiodegradable polymer matrices to manufacture jute FRC that are not fully green (100% bio-based) composites. The application of these bio-based nonbiodegradable composites has been increasing in the automotive and other manufacturing industries. Some green composites are manufactured using bio-based and biodegradable resin but the application of such materials is limited in the automotive and other structural applications, because of their poor mechanical properties. Efforts should be put into developing suitable bio-based and biodegradable resin for manufacturing green and environmentally sustainable high-performance natural FRCs. Such composites could potentially help reducing non-degradable plastic waste and improve the overall carbon footprints associated with composites industries. Based on the articles discussed in this review, the researchers should pay attention toward the development of the next generation of high-performance green composites by incorporating the graphene and other 2D materials in the jute fiber reinforced composite.
